# Analysis of COVID-19 pandemic impact on the presenting complaints of the emergency department visits

**DOI:** 10.1097/MD.0000000000028406

**Published:** 2021-12-23

**Authors:** Yen-Wen Lai, Ching-Tang Hsu, Yu-Ting Lee, Wei-Lung Chen, Jiann-Hwa Chen, Chien-Cheng Huang, Jui-Yuan Chung

**Affiliations:** a Department of Emergency Medicine, Cathay General Hospital, Taipei, Taiwan; b Fu Jen Catholic University School of Medicine, Taipei, Taiwan; c Department of Emergency Medicine, Chi-Mei Medical Center, Tainan, Taiwan; d Department of Environmental and Occupational Health, College of Medicine, National Cheng Kung University, Tainan, Taiwan; e Department of Senior Services, Southern Taiwan University of Science and Technology, Tainan, Taiwan.

**Keywords:** coronavirus disease 2019, emergency department, emergency visits, presenting complaints

## Abstract

The impact of coronavirus disease 2019 (COVID-19) on economic and medical systems is significant, especially in the emergency department (ED). The patterns of ED visits have also changed significantly and may play a crucial role in rearranging medical resources to the most needed departments during the pandemic.

This was a retrospective study conducted in hospitals of the Cathay Health System. All patients presented to the EDs between January 21, 2020 to April 30, 2020 (pandemic stage) and January 21, 2019 to April 30, 2019 (before the pandemic stage). Basic demographics, including visit characteristics, disposition, and chief complaints, of the patients visiting the ED between these 2 periods of time will be compared and analyzed.

A total of 71,739 patients were included in the study. A reduction in ED visits was noted in 15.1% (32,950 ED visits) during the pandemic stage. ED visiting patients with the chief complaints of upper respiratory infection and social problems increased by 14.23% and 1.86%, respectively, during the pandemic period. Critical chief complaints such as cardiac arrest, chest pain and altered mental status decreased to less than the ED visits difference (−15.1%) between the pandemic and prepandemic stages, for 0%, −7.67%, and −13.8% respectively.

Rearrangement of the ED pediatric staff to the COVID-19 special units and recruiting more social workers to the ED should be performed to respond to the COVID-19 pandemic.

## 1 Introduction

Severe acute respiratory syndrome coronavirus 2 is a highly contagious novel virus that may result in both critical and fatal conditions. The virus was transmitted by droplets or contaminated hands, with an incubation period of up to 14 days. Upper respiratory infection symptom signs are frequently presented, while timely diagnosis, quarantine, and supportive treatment are crucial to further treat these patients.^[1]^

In late December 2019, the first cluster of coronavirus disease 2019 (COVID-19) was reported in Wuhan, China. A few months later, the COIVD-19 virus spread worldwide, and Taiwan was no exception. On January 21, 2020, the first imported case of COVID-19 was reported in Taiwan.^[2]^ On February 28, 2020, the first in-hospital COVID-19 cluster infection occurred, and the peak pandemic period commenced. The number of COVID-19 patients rapidly increased from March to April 2020. It was not until December 19, 2020, when the number of confirmed COVID-19 cases in Taiwan reached 759.^[3]^

The impact of COVID-19 on economic and medical systems is significant, especially in the emergency department (ED). In contrast to the assumed condition of overcrowded health care systems, statistics from other countries have shown a reduction in ED patient volume during the COVID-19 pandemic.^[4,5]^ The patterns of ED visits have also changed significantly during the pandemic season, with decreased ED visits in non-COVID-19 diseases, and an increased visit rates of out-of-hospital cardiac arrests, probably due to delay in seeking medical attention.^[6]^ However, the impact of the altered trend of ED visiting patients on ED physician workload and ED medical resources arrangement were less understood.

We therefore conducted this study to evaluate the impact of the COVID-19 pandemic on the number of ED visiting patients, mode of arrival, triage level, disposition, time of visit, mode of arrival, and chief complaints, which may benefit in allocating medical resources effectively to the most needed departments, and reduce the possibility of overcrowding in the healthcare system during the pandemic.

## 2 Methods

### 2.1 Study setting

This was a retrospective study conducted in the hospitals of the Cathay Health System, including a medical center, a regional hospital, and a district hospital in northern Taiwan. One of the study hospitals is an urban medical center with an 800-bed capacity and an estimated total annual ED visit volume of 60,000. The other 2 included hospitals are both located in rural areas with capacities of 642-bed and 348-bed. The estimated annual ED visiting volumes were 48,000 and 30,000. Patients visiting the ED of these 3 hospitals between January 21, 2020 to April 30, 2020 and January 21, 2019 to April 30, 2019 will be recruited.

### 2.2 Study design

Patient information will be extracted from the electronic medical record (EMR) system and further divided into the “during pandemic” group, January 21, 2020 to April 30, 2020; and the “before pandemic” group, January 21, 2019 to April 30, 2019. The pandemic period was set up by the first confirmed case in Taiwan on January 21, 2020 and ended on April 30, 2020, which was the 4^th^ day after no confirmed cases for 3 consecutive days. All patients, including children and adults who presented to the EDs, were considered eligible for recruitment between the 2 periods. Missing or duplicated data were excluded to ensure the integrity of the information.

### 2.3 Variables

Basic demographics, including visit characteristics, disposition, and chief complaints of the patients visiting the ED during these 2 periods of time, will be obtained. Visiting characteristics consisted of total daily visits, mode of arrival, and time of visit; chief complaints were based on patients’ narratives recorded on the EMR and sorted into 33 common discomforts. Patients were triaged to different sections based on a combination of the Canadian Triage and Acuity Scale and clinical criteria. All triage nurses received formal training and had more than 1-year of ED working experience.

### 2.4 Ethical statement

The present study was approved by the Institutional Review Board of Cathay General Hospital and was conducted following the Declaration of Helsinki. The need for informed consent from the patients was waived, as this was an observational study.

### 2.5 Statistical analysis

Our data were analyzed using IBM SPSS Statistics for Windows (version 25.0). Categorical variables were presented as numbers and percentages, while normally distributed continuous variables were presented as mean ± standard deviation. The chi-square test was used to analyze the categorical variables, and an independent t-test was used to analyze the normally distributed continuous variables. Incidence rates were calculated as the number of results of interest divided by the total number of ED visits in each period. Incidence rates were presented as the frequency of the results per 100 ED visits. We also calculated the percentage difference in the number of chief complaints between the “before pandemic” period and the “during pandemic” period.

## 3 Result

**Figure 1:**
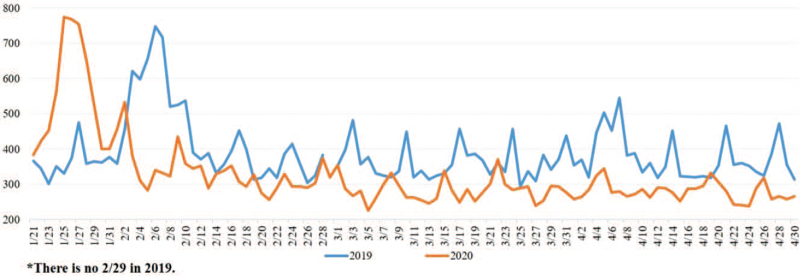
Comparison of total ED volume between the 2 periods.

A total of 71,739 patients were included in this study. There were 38,789 ED visits before the pandemic period between January 21, 2019 and April 30, 2019; a reduction in ED visits was noted for 15.1% (32,950 ED visits) during the pandemic period (Fig. 1). The sex ratio showed a female percentage of 52.6% and 51.5%, both before and during the pandemic period, which was significantly higher than the male percentage of 47.4% and 48.5%, respectively (Table 1).

**Table 1:** Comparisons of demographic characteristics of ED visits between the 2 time periods.

Characteristics	Before pandemic (January 2019–April 2019) (N = 38,789)	During pandemic (January 2020–April 2020) (N = 32,950)	Difference (%)	*P*-value
Total visits/day – no. (SD)	387.89 (86.07)	326.30 (103.94)	−15.88	<.001
Age – no. (%)				
Age < 18	6009 (15.5)	4107 (12.5)	−31.65	<.001
18 ≤ Age < 65	23,480 (60.5)	21,488 (65.2)	−8.48	
Age ≥ 65	9300 (24.0)	7355 (22.3)	−20.91	
Sex – no. (%)				
Male	18,373 (47.4)	15,987 (48.5)	−12.99	.002
Female	20,416 (52.6)	16,963 (51.5)	−16.91	
Mode of arrival^∗^ – no. (%)				
Walk-in	28,035 (75.6)	24,224 (76.9)	−13.59	<.001
Wheelchair	3727 (10.1)	2932 (9.3)	−21.33	
Ambulance	3771 (10.2)	3398 (10.8)	−9.89	
Being held	1533 (4.1)	956 (3.0)	−37.64	
Triage – no. (%)				
Triage 1	812 (2.1)	723 (2.2)	−10.96	<.001
Triage 2	5299 (13.7)	4228 (12.8)	−20.21	
Triage 3	29,308 (75.6)	24,502 (74.4)	−16.40	
Triage 4	3036 (7.8)	3137 (9.5)	3.33	
Triage 5	334 (0.9)	360 (1.1)	7.78	
Disposition – no. (%)				
Admission	5434 (14.0)	4444 (13.5)	−18.22	<.001
Discharge	32,279 (83.2)	27,687 (84.0)	−14.23	
AMA	784 (2.0)	540 (1.6)	−31.12	
Transfer	185 (0.5)	146 (0.4)	−21.08	
Mortality	107 (0.3)	133 (0.4)	24.30	
Time of visit – no. (%)				
Early morning (00.00–08.00)	7307 (18.8)	5680 (17.2)	−22.25	<.001
Day time (08.00–17.00)	16,697 (43.0)	14,947 (45.4)	−10.46	
Nighttime (17.00–24.00)	14,785 (38.1)	12,323 (37.4)	−16.65	

AMA = against medical advice, SD = standard deviation.

∗ Some records missed the mode of arrival.

The mean daily ED visiting patients decreased prominently from 387.89 ± 86.07 patients per day (before the pandemic) to 326.30 ± 103.94 patients per day (during the pandemic period). Compared to the 15.5% pediatric (age < 18) and 24.0% elderly (age ≥ 65) ED visiting patients before the pandemic period, significant reductions in pediatric and elderly ED visiting patients were noted for 12.5% and 22.3%, respectively, during the pandemic period. The usage of the emergency medical services (EMS) system (ambulance) slightly increased during the pandemic period by 10.8%, compared to 10.2% before the pandemic period. A higher percentage of triage level 4 and 5 patients were seen in the pandemic period for 9.5% and 1.1%, and 7.8% and 0.9%, respectively, before the pandemic period. More patients (84.0%) were discharged during the pandemic period than before the pandemic period (83.2%) (Table 1).

Generally, most ED visiting chief complaints decreased during the pandemic period, compared to the prepandemic period. However, ED visiting patients with the chief complaints of upper respiratory infection and social problems increased by 14.23% (*P*-value < .01) and 1.86% (*P*-value = .12), respectively, during the pandemic period. Critical chief complaints, such as chest pain and altered mental status decreased to less than the ED visits difference between pandemic and prepandemic (15.1%) for −7.67% (*P*-value < .01) and −13.08% (*P*-value = .34) respectively. Meanwhile, combined the number of patients transferred from OPD, LMD, and hospitals, the percentage decreased by 7.84% compared to the prepandemic period (Table 2).

**Table 2:** Chief complaints of total ED visits between periods.

Chief Complaints	Before pandemic (January 2019–April 2019) (N = 38,789)		During pandemic (January 2020–April 2020) (N = 32,950)		Difference (%)	*P*-value
	N	Incidence	N	Incidence		
Fever	5762	14.85	5696	17.29	−1.14	<.01
URI	4220	10.88	4823	14.63	14.23	<.01
Cellulitis	717	1.85	570	1.73	−20.50	.44
Abdominal pain	5497	14.17	4421	13.42	−19.57	.49
AGE symptoms	4715	12.16	2448	7.43	−48.05	<.01
Constipation	172	0.44	95	0.29	−44.77	<.01
GIB symptoms	451	1.17	314	0.95	−30.38	<.01
Colorectal problems	63	0.16	58	0.18	−7.94	.26
Chest pain	1916	4.94	1769	5.37	−7.67	<.01
Hypertension	422	1.09	219	0.66	−48.10	<.01
Shortness of breath	1758	4.53	1472	4.47	−16.27	.18
Dizziness	2585	6.67	1559	4.73	−39.69	<.01
Headache	1140	2.94	613	1.86	−46.23	<.01
Convulsion	160	0.41	122	0.37	−23.75	.54
Stroke symptoms	293	0.76	247	0.75	−15.70	.77
Altered mental status	367	0.95	319	0.97	−13.08	.34
Malaise	432	1.11	272	0.83	−37.04	<.01
Myalgia	2185	5.63	1524	4.63	−30.25	<.01
Glycemic problems	186	0.48	148	0.45	−20.43	.96
Urological symptoms	1152	2.97	882	2.68	−23.44	.49
Medical tube, probe, and catheter problems	641	1.65	478	1.45	−25.43	.42
Trauma	8641	22.28	7326	22.23	−15.22	<.01
Facial feature problems^∗^	1009	2.60	686	2.08	−32.01	<.01
OBS-GYN related problems	748	1.93	671	2.04	−10.29	<.01
Dermatology problems	1017	2.62	658	2.00	−35.30	<.01
Cardiac arrest	126	0.32	126	0.38	0	.16
Transfer from OPD and LMD	386	1.00	251	0.76	−34.97	<.01
Transfer from hospital	990	2.55	1017	3.09	2.73	<.01
Psychological problems	268	0.69	235	0.71	−12.31	.38
Social problems^†^	161	0.42	164	0.50	1.86	.12
Ask for screening	/	/	84	0.25	/	
Suggest screening by TCDC	/	/	115	0.35	/	
Others	308	0.79	190	0.58	−38.31	<.01

N represents the raw number of cases in each period. Incidence is the number of results of interest divided by the total ED visits in each period, reported as the frequency of the outcome per 100 ED visits. The difference compares the raw number of cases between the before and during pandemic periods, presented as percentages. AGE = acute gastroenteritis, GIB = gastrointestinal bleeding, LMD = local medical department, OBS-GYN = obstetrics-gynecology, OPD = outpatient department, TCDC = Taiwan Centers for Disease Control, URI = upper respiratory infection.

∗ Facial feature problems, including eyes, ear, nose, and throat and dental problems.

† Social problems include family violence and sexual assault.

## 4 Discussion

Recent studies have shown a significant reduction in ED visits during the first few weeks of the pandemic.^[7]^ Similarly, we found a substantial decrease in total ED visits in all Cathay Health System hospitals during the pandemic period. Several factors, including social distancing policies, school closures, and media influence, may decline total ED visits.^[8,9]^ This circumstance was especially observed among the vulnerable populations: pediatric and older patients, as parents and caregivers were reluctant to bring their children or elderlies to the hospitals in fear of being infected by COVID-19 during the ED visit. Rearrangement of the pediatric department staff to the COVID-19 special units may be performed to properly utilize medical resources during the pandemic.

According to our research, increased ED arrival via ambulance was noted during the pandemic period. A study of EMS showed that the COVID-19 pandemic was associated with negative collateral health effects; however, they found no evidence that people were reluctant to call an ambulance when they experienced critical symptoms, such as stroke or heart attack.^[11]^ Similarly, in the present study, the proportion of patients who were triaged as level 1 slightly increased during the pandemic stage compared to the prepandemic stage. Furthermore, no difference was noted over the ED visiting chief complaint of cardiac arrest before and during the pandemic. This indicates that people would still visit the ED by activating the EMS, if necessary.

According to the chief complaints of total ED visits between the 2 periods: Firstly, it is ambiguous to conclude that the lower general ED visits observed in our study resulted from the actual low incidence of illness, injury, or care shifted to other local medical clinics. However, a significant reduction of 15.2% (*P*-value < .01) in trauma patients was noted in our study. This indicates less social events and less traffic mobility during the pandemic period, either due to social distance or school closure policies. A study in Spain discovered a significant decrease in the number of accidents by 74.3% during the pandemic period, compared to the prepandemic period. This study further concluded that the reduction in mobility may result in a decline in accidents and injuries.^[9]^

Secondly, as the classic presentation of COVID-19 is upper respiratory tract infection symptoms, such as fever, sore throat, fatigue, cough, or dyspnea,^[1]^ ED visiting patients with the chief complaint of upper respiratory tract infection increased dramatically during the pandemic period, probably because of the fear of being infected by the COVID-19 virus^[10]^. These circumstances also increase the number of patients with low triage acuity levels of 4 or 5 during the pandemic period. Similarly, we also observed a significant decline in the percentages of less urgent and non-COVID-19 related ED visiting chief complaints, such as hypertension, acute gastroenteritis, constipation, and dizziness, which were noted for 48.10%, 48.05%, 44.77%, and 39.69% during the pandemic stage, compared to the prepandemic period.

Thirdly, reductions in the percentages of substantially higher urgency ED visiting chief complaints (less than the ED visits difference between pandemic and prepandemic of 15.1%), such as altered mental status (−13.08%, *P*-value < .34) and chest pain (−7.67%, *P*-value < .01), were also observed during the pandemic stage. These circumstances may be related to the lockdown policies and less social activities during the pandemic, which could further consequence in less trigger factors of vascular diseases, including physical activity, air pollution, or work-related stress.^[12]^

Finally, although not significant, an increased proportion of social problems was noted during the pandemic, compared to the prepandemic period in the current study. A similar result was noted in a study conducted in China, which reported that the prevalence of psychological and social problems had increased during the COVID-19 pandemic. A possible reason was assumed to be related to frequent social medical exposure.^[13]^ Meanwhile, the “stay at home” policy during the pandemic period may lead to an increase in domestic violence, as the home is often an unsafe place for these victims.^[14]^ Therefore, more social workers should be recruited to the ED to respond to such circumstances.

Our study has several limitations. First, this was a retrospective study that utilized the EMR system between 2019 and 2020. Hence, some important data could not be obtained. Second, although 3 different hospitals were included in this study, all hospitals were located in northern Taiwan, and further study is needed to validate our results. Finally, individual bias may occur in different triage nurses while triaging patients, although all triage nurses are required to receive formal triage training.

## 5 Conclusion

A significant decline in the number of pediatric ED visits and an increasing proportion of social problem visits were observed during the COVID-19 pandemic. According to the results of this study, rearrangement of the ED pediatric staff to the COVID-19 special units and recruiting more social workers to the ED should be performed to respond to the COVID-19 pandemic.

## Author contributions

JYC and CCH designed the study; YWL, CTH, YTL, and JYC wrote the manuscript. JYC performed statistical analyses. The JHC, WLC, and CCH provided professional suggestions. All authors have read and approved the final manuscript.

**Conceptualization:** Chien-Cheng Huang, Jui-Yuan Chung.

**Methodology:** Jui-Yuan Chung.

**Supervision:** Wei-Lung Chen, Jiann-Hwa Chen, Chien-Cheng Huang.

**Writing – original draft:** Yen-Wen Lai, Ching-Tang Hsu, Yu-Ting Lee.

**Writing – review & editing:** Jui-Yuan Chung.
